# Minimal Clinically Important Differences in Inspiratory Muscle Function Variables after a Respiratory Muscle Training Programme in Individuals with Long-Term Post-COVID-19 Symptoms

**DOI:** 10.3390/jcm12072720

**Published:** 2023-04-05

**Authors:** Tamara del Corral, Raúl Fabero-Garrido, Gustavo Plaza-Manzano, César Fernández-de-las-Peñas, Marcos José Navarro-Santana, Ibai López-de-Uralde-Villanueva

**Affiliations:** 1 Department of Radiology, Rehabilitation and Physiotherapy, Faculty of Nursing, Physiotherapy and Podiatry, Universidad Complutense de Madrid (UCM), 28040 Madrid, Spain; tamaradelcorral@gmail.com (T.d.C.); rfabero@ucm.es (R.F.-G.); marconav@ucm.es (M.J.N.-S.); ibai.uralde@gmail.com (I.L.-d.-U.-V.); 2 Instituto de Investigación Sanitaria del Hospital Clínico San Carlos (IdISSC), 28040 Madrid, Spain; 3 Department of Physical Therapy, Occupational Therapy, Rehabilitation and Physical Medicine, Universidad Rey Juan Carlos, 28922 Alcorcón, Spain; cesar.fernandez@urjc.es; 4 Cátedra Institucional en Docencia, Clínica e Investigación en Fisioterapia, Terapia Manual, Punción Seca y Ejercicio Terapéutico, Universidad Rey Juan Carlos, 28922 Alcorcón, Spain

**Keywords:** SARS-CoV-2, minimal clinically important difference, inspiratory muscle training, responsiveness

## Abstract

Objective: To establish the minimal clinically important difference (MCID) for inspiratory muscle strength (MIP) and endurance (IME) in individuals with long-term post-COVID-19 symptoms, as well as to ascertain which of the variables has a greater discriminatory capacity and to compare changes between individuals classified by the MCID. Design: Secondary analysis of randomised controlled trial of data from 42 individuals who performed an 8-week intervention of respiratory muscle training programme. Results: A change of at least 18 cmH_2_O and 22.1% of that predicted for MIP and 328.5s for IME represented the MCID. All variables showed acceptable discrimination between individuals who classified as “improved” and those classified as “stable/not improved” (area under the curve ≥0.73). MIP was the variable with the best discriminative ability when expressed as a percentage of prediction (Youden index, 0.67; sensitivity, 76.9%; specificity, 89.7%). Participants classified as “improved” had significantly greater improvements in quality of life and lung function compared with the participants classified as “stable/not improved”. Conclusion: In individuals with long-term post-COVID-19 symptoms, the inspiratory muscle function variables had an acceptable discriminative ability to assess the efficacy of a respiratory muscle training programme. MIP was the variable with the best discriminative ability, showing better overall performance when expressed as a percentage of prediction.

## 1. Introduction

Infection with severe acute respiratory syndrome coronavirus 2 (SARS-CoV-2) is responsible for the coronavirus disease 2019 (COVID-19) pandemic, which has resulted in millions of deaths and has put a major strain on health systems worldwide. Although most patients recover spontaneously or after acute-phase management, clinicians are now faced with treating long-term post-COVID-19 symptoms [1]. The most commonly reported persistent symptoms include fatigue, dyspnoea, sleep disorder, and myalgia in up to 41%, 31%, 30%, and 22% of cases, respectively, after more than 1 year of follow-up [2], all of which encourage sedentary lifestyles, induce limited exercise tolerance, and cause considerable deterioration of health-related quality of life (HRQoL) [1]. As recently reported, individuals with long-term post-COVID-19 symptoms can experience respiratory muscle dysfunction [3]. Individuals who have recovered from COVID-19 also exhibit depressed exercise tolerance and an exaggerated hyperventilatory response during exercise [1,4]. These symptoms might be associated with diaphragm fatigue and an increase in the concentration of metabolites that activate the so-called “metaboreflex”, causing a peripheral limit to exercise tolerance, characterised by a diffusion defect in oxygen delivery [4,5].

Clinical studies have often reported treatment effects as a change in the outcome measure supported by a measure of variability; however, a statistically significant change might not indicate a clinically meaningful change. There is growing acceptance of the importance of assessing the clinical benefit from the patient’s perspective, as well as establishing the outcome measure’s ability to detect clinical change and to determine ways to interpret the magnitude of the observed change [6]. The minimal clinically important difference (MCID) was therefore developed to add clinical relevance or patient experience to the reporting of an outcome measure. The MCID is defined as “the smallest difference in score which patients perceive as beneficial and which would mandate a change in the patient’s management” [7] and is useful because it links the magnitude of change with treatment decisions in clinical practice and emphasises the primacy of the patient’s perception [8]. The MCID of relevant outcomes of respiratory muscle training programs has been established, including the maximal inspiratory pressure (MIP) [9,10], inspiratory muscle endurance (IME) [10], and functional exercise tolerance measured by field tests [11,12,13,14] in patients with chronic obstructive pulmonary disease (COPD). Unfortunately, the MCID has not been determined for inspiratory muscle function variables in individuals with long-term post-COVID-19 symptoms. Therefore, the improvements in inspiratory muscle function as a primary outcome in clinical trials for this population remain difficult to interpret. The MCID could help clinicians not only assess whether improvements in inspiratory muscle function are clinically meaningful but also interpret the contribution of changes in muscle strength and endurance to improvement in relevant outcomes (e.g., HRQoL, exercise tolerance, peripheral muscle strength, and lung function) after a respiratory muscle training programme in individuals with long-term post-COVID-19 symptoms.

Thus, the primary aim of this study was to establish the MCIDs for the inspiratory muscle function variables (muscle strength and endurance) in individuals with long-term post-COVID-19 symptoms. The secondary objectives were to ascertain which of the inspiratory muscle function variables has a greater discriminatory capacity and to compare changes in HRQoL, exercise tolerance, peripheral muscle strength, and lung function between individuals who exceed the MCID and those who do not.

## 2. Materials and Methods

### 2.1. Study Design

The study consisted of a secondary analysis of data from a previously conducted randomised controlled trial (registered in the United States Clinical Trials Registry: NCT04734561) [15]. This randomised controlled trial was a parallel 4-arm, double-blinded study, and it followed the Consolidated Standards of Reporting Trials guidelines. Participants were randomised into one of the four interventions: (1) inspiratory muscle training; (2) respiratory muscle training (inspiratory and expiratory); (3) sham inspiratory muscle training; or (4) sham respiratory muscle training. The training was 40 min/day, split into two 20 min sessions (morning and afternoon), 6 times per week, over 8 weeks. Clinical assessments were performed at baseline and at 4 and 8 weeks.

For this secondary analysis, data from the 2 real training groups were pooled. In addition, participant data were only included if they had completed their baseline and 8-week assessments. Thus, a total of 42 individuals with long-term post-COVID-19 symptoms were analysed. For a paired 2-tailed *t*-test with an α of 0.05, a power of 0.80, and an expected effect size of at least 0.5 (a criterion considered by Cohen as the minimum effect size to detect clinically relevant differences) [16], the estimated sample size was 34 individuals. The effect size for respiratory muscle function outcomes could be even larger according to previous studies conducted on other respiratory disease [10], which would imply a slightly smaller sample. Consequently, the analysis of 42 individuals could be considered acceptable if the study were designed with the intention of establishing the MCID for respiratory muscle function outcomes.

### 2.2. Participants

COVID-19 survivors 18 years of age and older were included in the trial if they presented persistent post-COVID-19 symptoms of fatigue and dyspnoea for at least 3 months after the COVID-19 diagnosis had been confirmed. Candidates were excluded if they (1) presented a diagnosis of progressive respiratory, neuromuscular, or neurological disorders and/or psychiatric or cognitive conditions that hindered their ability to cooperate; (2) presented any contraindication for respiratory muscle training treatment; (3) lacked Internet access; or 4) had been previously included in a rehabilitation programme for their post-COVID-19 symptoms.

### 2.3. Outcome Measures

- Inspiratory muscle function: Inspiratory muscle strength was assessed by the MIP using a digital mouth pressure meter (MicroRPM; Carefusion, San Diego, CA, USA), according to the American Thoracic Society/European Respiratory Society (ATS/ERS) guidelines [17]. Three trials were performed with a difference of less than 10% between them; the highest value was recorded. The estimated inspiratory muscle strength values were established following the reference equation for the adult population [18]. Inspiratory muscle endurance was measured during a constant load breathing test using the POWERbreathe KH1 device (POWERbreathe International Ltd., Southam, UK), following the instructions established in a previously published protocol [19]. Participants breathed against a submaximal inspiratory load (55% MIP at baseline) until reaching an endpoint limited by their symptoms or their inability to breathe successfully against the load. The length of time for which participants were able to breathe against this load was recorded.
- Health-related quality of life: To measure HRQoL, we employed the EuroQol-5D questionnaire (EQ-5D-5L) [20], which consists of 5 dimensions with 5 response options based on severity level, ranging from 1 to 5. An index score was provided, ranging from 0 (death) to 1 (full health). Participants rated their current overall health on a visual analogue scale, ranging from 0 (poorest imaginable health) to 100 (best imaginable health).
- Exercise tolerance: Cardiorespiratory fitness was assessed by the Ruffier test [21], consisting of 30 squats in 45 s, with a tempo set by a metronome (80 beats per min). Heart rate (HR) was measured after 1 min of resting (HR_0_), immediately after completing the 30 squats (HR_1_), and after a 1 min recovery (HR_2_). Cardiorespiratory fitness was calculated using the following index: ((HR_0_ + HR_1_ + HR_2_) − 200)/10. Cardiorespiratory fitness correlates with HR due to HR at rest is a general indicator of wellness, while a decline in the HR response to submaximal exercise represents an enhancement in endurance. The linearity of the HR and oxygen consumption relation has been used to predict maximal oxygen uptake in submaximal tasks [22].
- Peripheral muscle strength: Lower-limb muscle strength was determined using the 1 min sit-to-stand (1-min STS) test according to a standardised protocol [23]. The number of times the participant gently touched the chair with their buttocks in 1 min, without using hands or arms to assist the movement, was recorded. Upper limb muscle strength (handgrip force) was assessed using a hand-held dynamometer (Jamar, Patterson Medical, IL, USA) [24]. Three measurements were performed for each hand, alternating sides, and the highest value was recorded.
- Lung function: Pulmonary function testing was assessed using a portable spirometer (Spirobank II USB, MIR, Rome, Italy), according to ATS/ERS guidelines [25]. Measurements included forced vital capacity (FVC), forced expiratory volume in the first second (FEV_1_), and their ratio (FEV_1_/FVC).

### 2.4. Anchor Outcome

After the respiratory muscle training, the participants (blinded to the results of their post-training assessments) completed the Global Rating of Change (GROC) scale [7], which was employed as an anchor variable for determining MCID. The GROC consists of a 15-point ordinal scale ranging from –7 (“a great deal worse”) to 7 (“a great deal better”). The participants were asked to rate the perceived change in their overall health since the start of the training by answering the following question using the GROC: “Compared with the first assessment/visit, how much change do you perceive in your overall health status after respiratory muscle training (including performance of activities of daily living, efforts/fatigue, and/or dyspnoea)?”

### 2.5. Data Analysis

The data analysis was performed using SPSS version 27.0 (SPSS Inc., Chicago, IL, USA). For all analyses, the statistical significance was set at 5% (*p* < 0.05).

The change in inspiratory muscle function in the whole sample was assessed using parametric tests, given that a normal distribution of the variables was assumed based on the results of the assumption tests and the central limit theorem (due to the large sample size; N > 30) [26]. Thus, a dependent samples *t*-test was used to determine the differences between pre- and post-training outcomes. Effect sizes were calculated according to Cohen’s method: small (0.20–0.49), medium (0.50–0.79), or large (≥0.8) [27].

The MCID for improvement perceived by the individual was determined by using an anchor-based method. Concretely, the anchor-based approach was performed using a receiver operating characteristic (ROC) curve analysis. This approach used the anchor variable (external criterion; GROC scale) to determine the optimal cut-off for the respiratory muscle function variables that corresponded to the least misclassification for discriminating between individuals who had improved and those who were unchanged or deteriorated. To calculate the MCID, participants were dichotomised into 2 groups according to GROC scores: (1) stable/not improved (no change or minimal improvement): those who scored +3 or less and (2) improved: those who scored +4 or more. A cut-off of +4 has classically been considered to determine the MCID [7,28].

Group comparisons between individuals with and without a change greater than MCID in inspiratory muscle function outcomes were performed using non-parametric tests due to the sample size (sample size ≤ 16 individuals in the groups without exceeding the MCID). In addition, the Shapiro–Wilk test showed a non-normality distribution for almost half of the data. The Mann–Whitney U test was used to detect between-group differences in quality of life, exercise tolerance, peripheral muscle strength, lung function at baseline, post-training, and difference between pre- and post-values (Δpre-post). The Wilcoxon test was used to compare pre- and post-training results within each group. The magnitude of the differences was calculated using an r effect size: small (r < 0.3), medium (0.30–0.5), or large (>0.5) [29].

## 3. Results

The study sample consisted of 42 individuals with long-term post-COVID-19 symptoms (12 men and 30 women) with a mean age of 47.93 ± 8.84 years (height, 165.9 ± 7.7 cm; weight, 74.69 ± 16.51 kg; and body mass index, 27.13 ± 5.81 kg/m^2^). All participants completed more than 95% of the training sessions, and no adverse effects were reported during the respiratory muscle training programme. The mean symptom duration since diagnosis was 354.21 ± 77.56 days, and 13 (31%) participants required hospital admission of whom three required invasive mechanical ventilation. Most participants showed inspiratory and/or expiratory muscle weakness at baseline (n = 32 (76%); MIP and/or maximal expiratory pressure <80% of predicted). This loss of muscle strength could be associated to deconditioning as a result of prolonged inactivity due to hospitalized or quarantined at home. More than half of the participants had smoked at some time in their lives (smokers, 11 (26%); ex-smokers, 12 (29%)). However, only one (2%) participant had impaired lung function (FVC <80% of predicted).

### 3.1. Findings Related with Minimal Clinically Important Difference

The distribution of participant responses according to their GROC scores was “improved” in 69% and “stable/not improved” in 31% (no change (12%) or minimal improvement (19%)). After 8 weeks of a respiratory muscle training programme, a large and statistically significant increase in both inspiratory muscle strength (ΔMIP in cmH_2_O, 33.05 ± 18.99 (95% CI 27.13 to 38.97; *p* < 0.001; *d* = 1.43); ΔMIP in % of predicted, 31.72 ± 17.60 (95% CI 26.23 to 37.20; *p* < 0.001; *d* = 1.75)) and inspiratory muscle endurance (ΔIME in cmH_2_O, 272.64 ± 158.17 (95% CI 223.35 to 321.93; *p* < 0.001; *d* = 2.05)) was observed in the entire sample. Table 1 shows the descriptive statistics for the change in inspiratory muscle function variables for the group classified as “improved” and the group classified as “stable/not improved”, as well as the multiple comparisons between them.

**Figure 1:**
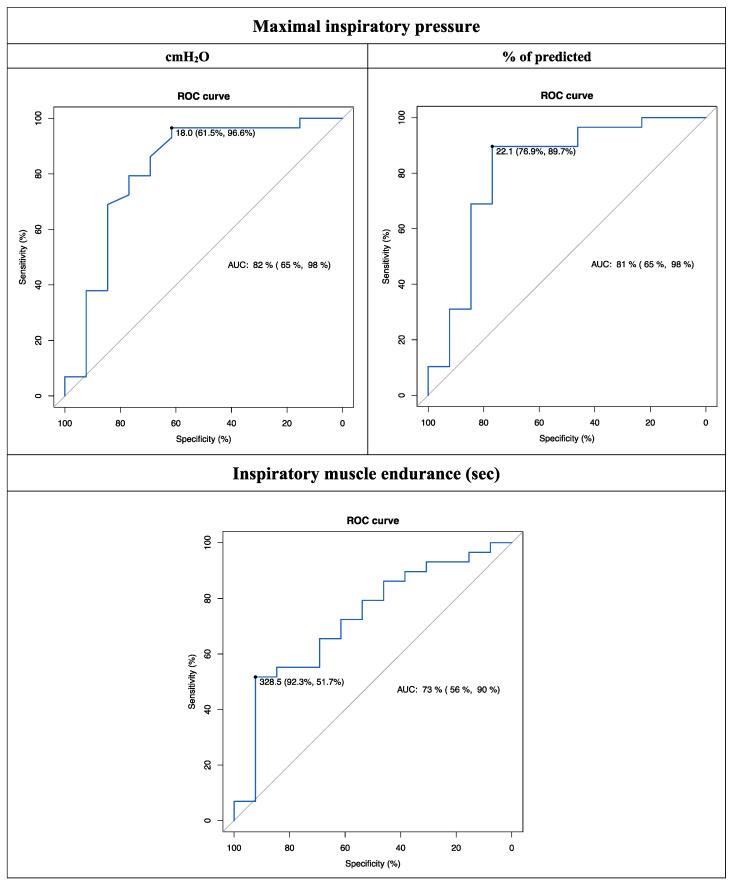
Receiver operating characteristic (ROC) curves for the inspiratory muscle function variables. Values expressed as: AUC (95% CI), area under the curve (95% confidence interval); MCID (Sen, Spe), minimal clinically important difference (sensitivity, specificity).

The ROC analysis results for the inspiratory muscle function variables are presented in Table 2. According to the ROC analysis, all variables showed acceptable discrimination between individuals who classified themselves as “improved” and those who classified themselves as “stable/not improved”, obtaining an AUC ≥0.73 (Figure 1). MIP was the variable with the best discriminative ability, showing better performance when expressed as a percentage of prediction (Youden index, 0.67) rather than in cmH_2_O (Youden index, 0.58). The ROC curve analysis established that a change of 18 cmH_2_O (sensitivity, 61.5%; specificity, 96.6%) or of 22.1% of that predicted (sensitivity, 76.9%; specificity, 89.7%) represents a meaningful clinical improvement in MIP. Thus, assuming 18 cmH_2_O or 22.1% of that predicted as MCID for MIP, 38.5% or 23.1% of the participants who classified themselves as “improved” were misclassified as “stable/not improved”, respectively.

### 3.2. Comparison between Individuals with and without a Change Greater Than MCID

Table 3 lists the descriptive statistics and multiple comparisons for the change in the assessed variables. The participants with a greater than MCID change in MIP, regardless of measurement unit, showed a medium/large and statistically significant increase in inspiratory muscle strength compared with those with a less than MCID change (*r* = 0.45–0.52). Similarly, the participants who exceeded MCID in IME showed a large and statistically significant increase in MIP compared with those with a change below MCID (MIP in cmH_2_O, *r* = 0.58; MIP in % of prediction, *r* = 0.62).

The participants with a change greater than the MCID set for the inspiratory muscle function variables (MIP and IME) showed a medium/large and statistically significant increase in HRQoL (*r* = 0.31–0.54) and FVC (*r* = 0.35–0.49) compared with those who did not exceed the MCID, except for the EQ-5D-5L index when the MCID for MIP was set at cmH_2_O (*p* = 0.182; *r* = 0.21). In addition, only the participants with a change above the MCID in MIP expressed as a percentage of prediction showed a medium and statistically significant increase in the FEV_1_/FVC ratio (*r* = 0.31) compared with those who did not exceed the MCID. There was no difference in exercise tolerance, peripheral muscle strength, or FEV_1_ between the participants who exceeded MCID in the inspiratory muscle function variables and those who did not. However, only the participants who exceeded MCID for MIP and IME showed a statistically significant improvement in exercise tolerance compared with their pre-training assessment.

## 4. Discussion

This study reports the first MCIDs for inspiratory muscle function variables in individuals with long-term post-COVID-19 symptoms after a respiratory muscle training programme. Using an anchor-based approach, our results indicate 18 cmH_2_O and 22.1% of predicted values as MCID for MIP, and 328.5 s as MCID for IME, suggesting that an increase over these values can be considered clinically relevant in this population. Furthermore, MIP was the variable with the best discriminative ability, showing better overall performance when expressed as a percentage of prediction due to the better metric properties detected. The MCID values presented here provide a way for clinicians to evaluate meaningful change in individual patients and for researchers to evaluate meaningful change between groups.

Both real training groups obtained significant improvements in inspiratory muscle strength and endurance after 8 weeks of a respiratory muscle training programme; these improvements could be considered clinically relevant, given that they were associated with large effect sizes (≥0.8). Differences were observed between the group classified as “improved” and the group classified as “stable/not improved”, with small to moderate effect sizes. Our results are supported by the study by McNarry et al. [30], who reported that an 8-week inspiratory muscle training programme could strengthen inspiratory muscles in individuals with self-reported COVID-19. Furthermore, participants classified as “improved” had significantly greater improvements in all inspiratory muscle function variables compared with the participants classified as “stable/not improved”. This underscores that patient-centred care requires careful and explicit consideration of the patient’s perspective to improve patient satisfaction [31].

The AUC ≥0.73 from all anchors demonstrated adequate discrimination ability to classify individuals who had undergone important changes from those who had not and therefore rendered this estimate for the MCID clinically useful. To our knowledge, no previous studies have established the MCID for inspiratory muscle function variables in individuals with long-term post-COVID-19 symptoms. We therefore discuss the results considering other respiratory conditions with similar features, while recognising that the differences in the population sample examined would yield larger MCID values. Our determination of the MCID value of 18 cmH_2_O for MIP is in line with the value of 17.2 cmH_2_O established for patients with COPD [9] and smaller than the MCID estimate by Gosselink et al. [10] of 13 cmH_2_O in the same population. With respect to IME, Gosselink et al. [10] reported that a change of at least 261 s was considered clinically significant; in our study, any change greater than 328.5 s was considered clinically important. The discrepancies observed between these studies could lie in the type of population studied in each investigation and by the fact that we used the anchor-based approach—a more conservative and exhaustive method—which is essentially based on the participant’s perceived improvement after an intervention and is therefore subjective. This is in contrast to the approach employed in the Gosselink et al. [10] study, which was based on mathematics (summary effect size), with no intervention performed, a better approach to estimate the minimal detectable change (MDC; the smallest change in score that can be detected beyond random error).

Following this argument, the MCID value for MIP reported by the current study could be considered a “real change” because it exceeded the recently redefined MDC of 17 cmH_2_O in moderate smokers [32]. Given that the MCID is an estimate of how much an outcome measure should change for that change to be considered “important”, this value should ideally be similar to or exceed the MDC value, so that the “important” change represented by the MCID also exceeds the value that is estimated to exceed the measurement error in an outcome measure [33]. It is important to note that the MCID value for a particular measure can vary depending on the clinical context and decision at hand, the baseline from which the patient starts, and whether they are improving or deteriorating [34]. Thus, the MCID should be judiciously applied to any particular clinical or research context.

In general terms, MIP expressed as a percentage of prediction was the value with the overall best discriminatory capacity because it assumes the best Youden index (0.67) and an optimal certainty threshold that balances false-negative rates. Specifically, the best balance between the positive and negative likelihood ratio (LR) was detected when the MCID for MIP was set at 22.1% of predicted value (LR+, 7.5; LR–, 0.3; sensitivity, 76.9%; specificity, 89.7%). This is in line with Decramer [35], who reported that MIP had been shown to correlate significantly, albeit weakly, with the response to training in patients with COPD, allowing this outcome to be used to predict the response to the rehabilitation programme and that can be used as a guideline for basing clinical decisions. Reinforcing the relevance of the MIP in detecting the individuals who improved, MIP expressed as cmH_2_O showed the highest LR+ (18.1) when a change of ≥18 cmH_2_O was produced, indicating a large likelihood of determining with greater certainty that the individual would feel clinically better if it exceeded that value. In contrast, the smallest LR— was observed for IME, suggesting that a change lower than 328.5 s assumes a large likelihood that the individual would not perceive clinical improvement. However, the LR+ for IME was trivial; we therefore consider that in clinical practice, it could be more useful to use the MCID established for MIP both in cmH_2_O and as a percentage of prediction. MIP expressed as a percentage of prediction is adjusted for anthropometric variables, which affect the results of MIP, thereby possibly explaining the slightly higher diagnostic accuracy over MIP expressed in cmH_2_O. This result is supported by the positive correlation of MIP with body composition found in patients with COPD [36] and in healthy individuals [37]. Thus, our results suggest that the use of the MCID for MIP expressed as a percentage of prediction should be the first measure of choice to identify whether a patient has experienced an improvement; also, the probability would increase substantially if we then verify that the change exceeds 18 cmH_2_O. Future studies are needed to reinforce or contradict our findings.

We were able to perform group comparisons between the participants with and without a change greater than MCID, which is one of the novelties and strengths of our study, reinforced by the fact that clinical improvements occurred not only in relation to inspiratory muscle function variables but also relative to HRQoL and FVC, making our results more clinically applicable. In fact, only the participants with a change above the MCID in MIP expressed as a percentage of prediction showed a statistically significant decrease in the FEV_1_/FVC ratio compared with those who did not exceed the MCID. For reasons beyond our knowledge, participants who did not exceed the MCID of MIP expressed as a percentage of prediction increased FEV_1_ without improving FVC. As a result of inspiratory muscle training, FVC is expected to improve due to an increase in inspired volume, so there would be a slight increase in FEV_1_ attributed to lung compliance. This trend occurred in all group comparisons of lung function variables between the participants with and without a change greater than MCID, except for MIP expressed as a percentage of prediction. In our opinion, this is the reason why these differences were statistically significant, but not clinically relevant (<2%). In addition, the results showed a non-significant trend towards an increase in exercise capacity and peripheral muscle strength, further reinforcing that this value is slightly higher relative to all reported MCIDs. There is a decompensation between the groups compared, with a higher proportion in the group that exceeded the MCID. Therefore, future studies comparing homogeneous groups are necessary.

This study presents some limitations. The study was derived from a randomised controlled trial that was not primarily designed to estimate the MCID of inspiratory muscle function variables; however, the sample size calculation performed for this new study was adequate for detecting clinically relevant differences. In contrast, the study was not designed to detect differences between individuals with and without a change greater than MCID in inspiratory muscle function variables. Another limitation was the small number of participants without a GROC change, which might have affected the accuracy of estimating the specificity of the cut-off. Lastly, the generalisability of these results is limited to individuals with long-term post-COVID-19 symptoms from a single metropolitan area with characteristics similar to those of this study’s sample. Caution should be used in generalising these current findings to patients in other settings with other characteristics, such as acute phase of infection, because the improvements could observed due to the progression of the disease itself.

This study had some clinical implications. The MCIDs reported by the current study may be used to enhance the interpretability and meaningfulness of changes in improvement scores derived from clinical trials that examine the efficacy of interventions designed to improve inspiratory muscle function in individuals with long-term post-COVID-19 symptoms. In addition, researchers could express the results in terms of the proportion of participants in the experimental group who exceeded the MCID values compared with the same proportion of participants in the comparison group, which could provide a more clinically relevant method for examining the differences between intervention strategies. These values can be used to assess the progress of individual patients from a clinical standpoint and to illustrate to patients, caregivers, and third-party payers that “important” change has taken place, which should be a guide for planning patient management.

## 5. Conclusions

The present study indicated that, in individuals with long-term post-COVID-19 symptoms, the inspiratory muscle function variables (MIP and IME) had an acceptable discriminative ability to assess the efficacy of a respiratory muscle training programme. Specifically, a change of at least 18 cmH_2_O and 22.1% of the predicted value for MIP and 328.5 s for IME represented the MCID for judging clinical change in inspiratory muscle function. MIP was the variable with the best discriminative ability, showing better overall performance when expressed as a percentage of prediction. Individuals with a change greater than the MCID established for inspiratory muscle function variables showed a statistically significant increase in quality of life and lung function compared with those who did not exceed the MCID.

## Figures and Tables

**Table 1:** Descriptive statistics and multiple comparisons between groups for change in inspiratory muscle function.

Outcome	Group	Mean ± SD; Median (IQR)			Within-Group Differences *p*-Value; *r* Effect Size
		Baseline	Post-Training	ΔPre-Post	
MIP (cmH_2_O)	Improved	78.45 ± 19.24 75 (64–94)	117.41 ± 26.3 117 (100.5–130)	38.97 ± 17.37 36 (28–50)	*p* < 0.001; *r* = 0.87
	Stable/not improved	97.92 ± 22 93 (82.5–113)	117.77 ± 20.47 114 (104.5–139)	19.85 ± 15.97 17 (11–26)	*p* < 0.001; *r* = 0.83
**Between-group differences for ΔPre-Post training** *p*-value; *r* effect size				*p* < 0.001; *r* = 0.50	
MIP (% pred)	Improved	74.95 ± 15.57 73.75 (66.63–87.09)	112.19 ± 20.87 114.10 (100.62–130.55)	37.23 ± 15.21 365.75 (24.34–52.54)	*p* < 0.001; *r* = 0.87
	Stable/not improved	91.4 ± 13.86 95.91 (80.24–100.77)	110.82 ± 16.21 111.61 (99.46–120.88)	19.42 ± 16.76 18.14 (9.74–21.76)	*p* < 0.001; *r* = 0.83
**Between-group differences for ΔPre-Post training** ***p*-value; *r* effect size**				*p* < 0.001; *r* = 0.50	
IME (sec)	Improved	200.17 ± 104.89 173 (117–286.5)	511.48 ± 151	311.31 ± 149.21 347 (225–428)	*p* < 0.001; *r* = 0.87
			494 (412–638)		
	Stable/not improved	166.23 ± 79.98 145 (113–182.5)	352.62 ± 128.23 343 (263.5–420.5)	186.38 ± 147.83 174 (85–291)	*p* < 0.001; *r* = 0.83
**Between-group differences for ΔPre-Post training** ***p*-value; *r* effect size**				*p* = 0.02; *r* = 0.36	

Abbreviatures: IME, inspiratory muscle endurance; IQR, interquartile range; MIP, maximal inspiratory pressure; SD, standard deviation.

**Table 2:** Receiver operating characteristic (ROC) analysis results for the inspiratory muscle function variables.

Outcome	MCID	AUC (95% CI)	Sensitivity	Specificity	Youden Index	LR+	LR−
MIP (cmH_2_O)	18	0.82 (0.65 to 0.98)	61.5	96.6	0.581	18.1	0.4
MIP (% pred)	22.1	0.81 (0.65 to 0.98)	76.9	89.7	0.666	7.5	0.3
IME (sec)	328.5	0.73 (0.56 to 0.90)	92.3	51.7	0.44	1.9	0.1

Abbreviatures: AUC, area under the curve; CI, confidence interval; IME, inspiratory muscle endurance; MCID, minimal clinically important difference; MIP, maximal inspiratory pressure; LR, likelihood ratio.

**Table 3:** Descriptive statistics and multiple comparisons for the change in variables assessed. Values are expressed as mean ± SD and median (IQR).

Outcome	Maximal Inspiratory Pressure (MCID = 18 cmH_2_O)		Maximal Inspiratory Pressure (MCID = 22.1% of pred.)		Inspiratory Muscle Endurance (MCID = 328.5 s)		Between-Group Differences *p*-Value; *r* Effect Size (a) MCID for MIP in cmH_2_O (b) MCID for MIP in % of pred. (c) MCID for IME in sec
	Did not exceed MCID	Exceeded MCID	Did not exceed MCID	Exceeded MCID	Did not exceed MCID	Exceeded MCID	
Inspiratory muscle function							
MIP (cmH_2_O)	— —	— —	— —	— —	26.31 ± 19.83 22.5 (14–34) ^a^	44 ± 11.15 49 (33.5–51) ^a^	(a) — (b) — (c) *p* < 0.001; *r* = 0.58
MIP (% pred)	— —	— —	— —	— —	23.23 ± 14.71 22.78 (14.53–28.67) ^a^	45.52 ± 12.57 51.36 (36.4–52.94) ^a^	(a) — (b) — (c) *p* < 0.001; *r* = 0.62
IME (sec)	116.56 ± 111.07 121 (60–174) ^b^	315.21 ± 142.3 310 (229–428) ^a^	168.23 ± 125.32 174 (85–250) ^a^	319.45 ± 150.24 347 (229–457) ^a^	— —	— —	(a) *p* < 0.001; *r* = 0.52 (b) *p* = 0.003; *r* = 0.45 (c) —
HRQoL							
EQ-5D-5L, index	0.145 ± 0.136 0.11 (0.049–0.214) ^a^	0.205 ± 0.172 0.22 (0.09–0.302) ^a^	0.12 ± 0.155 0.11 (0.023–0.214) ^a^	0.225 ± 0.162 0.23 (0.133–0.322) ^a^	0.152 ± 0.146 0.167 (0.023–0.253) ^a^	0.258 ± 0.178 0.254 (0.166–0.380) ^a^	(a) *p* = 0.182; *r* = 0.21 (b) *p* = 0.046; *r* = 0.31 (c) *p* = 0.039; *r* = 0.32
EQ-5D-5L, VAS	6.67 ± 7.5 5 (5–10) ^b^	20.67 ± 12.23 20 (14–28) ^a^	8.08 ± 11.46 5 (5–15) ^b^	21.97 ± 10.88 20 (15–28) ^a^	12.46 ± 9.82 14.5 (5–20) ^a^	26.12 ± 12.55 24 (17.5–35) ^a^	(a) *p* < 0.001; *r* = 0.51 (b) *p* < 0.001; *r* = 0.54 (c) *p* < 0.001; *r* = 0.50
Exercise tolerance							
Ruffier index	−0.62 ± 2.38 −1.2 (−2.2–−0.3)	−1.39 ± 2.72 −1.9 (−2.7–0.1) ^a^	−0.19 ± 2.34 −0.3 (−1.2–1.3)	−1.69 ± 2.67 −2.2 (−3–0) ^a^	−0.87 ± 2.12 −1.2 (−2.3–0.1) ^b^	−1.82 ± 3.32 −2.35 (−3.55–0.35) ^b^	(a) *p* = 0.154; *r* = 0.22 (b) *p* = 0.094; *r* = 0.26 (c) *p* = 0.200; *r* = 0.20
Peripheral muscle strength							
1 min STS (n of squats)	10.67 ± 9.57 11 (4–18) ^b^	12.52 ± 10.34 13 (7–17) ^a^	7.23 ± 11.4 6 (4–13) ^b^	14.31 ± 8.8 14 (9–17) ^a^	10 ± 10.19 10 (4–16) ^a^	15.56 ± 9.23 15.5 (11–20) ^a^	(a) *p* = 0.085; *r* = 0.27 (b) *p* = 0.058; *r* = 0.29 (c) *p* = 0.102; *r* = 0.25
Handgrip (Kg)	−1 ± 3.85 −1 (−4–−0.5)	1.21 ± 4.82 2 (−2.5–3)	−0.77 ± 3.53 −1 (−3.5–−0.5)	1.41 ± 5.02 2 (−2.5–3)	0.98 ± 5.43 −0.5 (−2.5–4)	0.34 ± 3.23 1.5 (−2.75–2.5)	(a) *p* = 0.262; *r* = 0.17 (b) *p* = 0.236; *r* = 0.18 (c) *p* = 0.876; *r* = 0.02
Lung function							
FVC (% pred)	−3.78 ± 10.32 −2 (−9–3)	5.27 ± 11.13 3 (0–9) ^a^	−0.77 ± 12.36 −2 (−6–3)	5.17 ± 10.77 3 (1–9) ^a^	0.5 ± 12.43 0 (−3–3)	7.94 ± 8.12 6 (2–10.5) ^a^	(a) *p* = 0.034; *r* = 0.33 (b) *p* = 0.025; *r* = 0.35 (c) *p* < 0.001; *r* = 0.49
FEV_1_ (% pred)	−1.33 ± 8.63 2 (−4–3)	2.76 ± 10.59 2 (−3–6)	1.92 ± 11.76 2 (−3–3)	1.86 ± 9.71 2 (−3–5)	0.88 ± 11.27 2 (−3–5)	3.5 ± 8.39 2.5 (−1–7.5)	(a) *p* = 0.282; *r* = 0.17 (b) *p* = 0.653; *r* = 0.07 (c) *p* = 0.364; *r* = 0.14
FEV_1_/FVC (%)	−0.67 ± 2.45 0 (−1–0)	−1.12 ± 3.19 −1 (−2–0) ^b^	0.31 ± 3.38 0 (−1–1)	−1.62 ± 2.7 −2 (−3–0) ^a^	−0.77 ± 3.3 −0.5 (−2–0)	−1.44 ± 2.56 −1.5 (−3–0) ^b^	(a) *p* = 0.377; *r* = 0.14 (b) *p* = 0.046; *r* = 0.31 (c) *p* = 0.295; *r* = 0.16

Abbreviatures: EQ-5D-5L, EuroQol-5D questionnaire; FEV_1_, force expiratory volume at 1st second; FVC, force vital capacity; HRQoL, Health-related quality of life; IQR, interquartile range; IME inspiratory muscle endurance; MCID, minimal clinically important difference; MIP, maximal inspiratory pressure; SD, standard deviation; STS, sit-to-stand; VAS, visual analogue scale; % pred, percentage of predicted value. ^a^ Statistically significant within-group differences from baseline values, *p* < 0.05, ^b^ Statistically significant within-group differences from baseline values, *p* < 0.01.

## Data Availability

Available upon request.
